# The Impact of Working Conditions in the Public Health Sector on the Mental Health of Young Doctors in Tunisia

**DOI:** 10.1192/j.eurpsy.2025.1573

**Published:** 2025-08-26

**Authors:** R. Nakhli, L. S. Chaibi, W. Dhaouadi, M. Bouchandira

**Affiliations:** 1 Occupational Medicine, Faculty Of Medicine of Sousse, Hachad University Hosiptal; 2 D Psychiatric department, Razi Hospital; 3 Faculty Of Medicine of Tunis, Tunis, Tunisia

## Abstract

**Introduction:**

Recent events in Tunisia have highlighted the severe challenges faced by young doctors, including high stress levels, burnout, and mental health issues.

**Objectives:**

This study aims to shed light on the often-invisible realities of the medical profession in Tunisia by exploring the impact of working conditions and health policies on the mental health of young doctors.

**Methods:**

This multicentric cross-sectional study collected data from 385 interns and residents working in four main university hospitals in Tunisia. The questionnaire included sections on sociodemographic characteristics, satisfaction with working conditions, burnout symptomatology (MBI), and anxiety and depressive symptomatology (HADS).

**Results:**

Most respondents (82.6%) were aged 25-29, with 73% being women. Most participants (83.1%) were residents. The study \revealed widespread dissatisfaction with working conditions, including remuneration (82% dissatisfied), days off and rest periods (73% dissatisfied), and overall working conditions (78% dissatisfied).

The average emotional exhaustion score was 31.883 (SD=10.2), indicating a high degree of burnout. Depersonalization scores averaged 10.176 (SD=6.3), and personal accomplishment scores averaged 23.838 (SD=8.7). The mean anxiety score was 8.776 (SD=4.2), and the mean depression score was 7.774 (SD=3.9), indicating moderate levels of anxiety and depressive symptoms.

**Image 1:**

**Figure:**
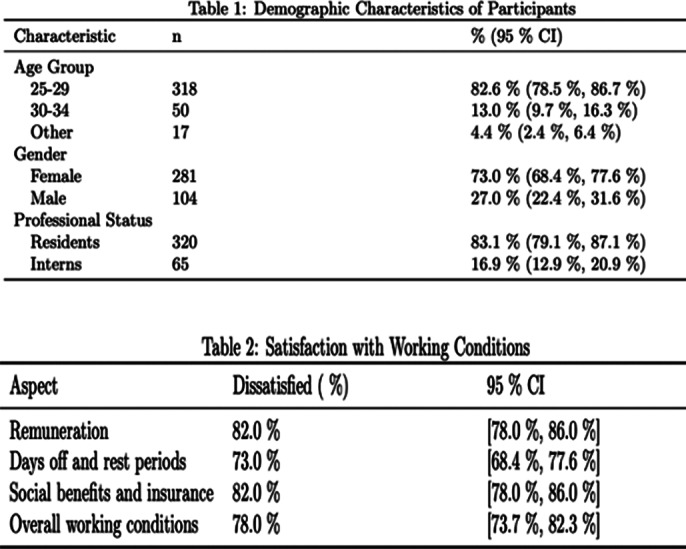

**Conclusions:**

The results paint a concerning picture of the mental health status of young doctors in Tunisia, with high levels of burnout, anxiety, and depression strongly correlated with poor working conditions and work-life imbalance. These findings highlight the urgent need for comprehensive reforms in the Tunisian healthcare system, including policy changes, organizational support, and improved medical education. Limitations include the cross-sectional nature of the study and potential self-reporting bias. Future longitudinal studies are needed to understand the long-term impacts of poor working conditions on physician mental health and career trajectories.

**Disclosure of Interest:**

None Declared

